# Genetically Engineered Foods and Moral Absolutism: A Representative Study from Germany

**DOI:** 10.1007/s11948-023-00454-0

**Published:** 2023-09-06

**Authors:** Johanna Jauernig, Matthias Uhl, Gabi Waldhof

**Affiliations:** 1 Center for the Philosophy of Freedom, University of Arizona, 1145 E South Campus Dr, Tucson, AZ 85721 USA; 2 Faculty of Computer Science, Technische Hochschule Ingolstadt, Esplanade 10, 85049 Ingolstadt, Germany; 3 Leibniz Institute of Agricultural Development in Transition Economies, Theodor-Lieser-Str. 2, 06120 Halle (Saale), Germany

**Keywords:** Genetic engineering (GE), Moral absolutism, Technology aversion, Moral convictions, GE debate, Consumer skepticism

## Abstract

There is an ongoing debate about genetic engineering (GE) in food production. Supporters argue that it makes crops more resilient to stresses, such as drought or pests, and should be considered by researchers as a technology to address issues of global food security, whereas opponents put forward that GE crops serve only the economic interests of transnational agrifood-firms and have not yet delivered on their promises to address food shortage and nutrient supply. To address discourse failure regarding the GE debate, research needs to understand better what drives the divergent positions and which moral attitudes fuel the mental models of GE supporters and opponents. Hence, this study investigates moral attitudes regarding GE opposition and support in Germany. Results show that GE opponents are significantly more absolutist than supporters and significantly less likely to hold outcome-based views. Furthermore, GE opponents are more willing to donate for preventing GE admission than supporters are willing to donate for promoting GE admission. Our results shed light on why the divide between opponents and supporters in the German GE debate could remain stark and stable for so long.

## Introduction

Environmental policy challenges, such as increasingly extreme weather conditions, has led to the new EU Commission’s stipulation that the European Union should become climate-neutral by 2050. This requires a profound transformation of agriculture, which can only be implemented with innovative, efficient, and sustainable technologies and processes. Consequently, the genetic engineering (GE) of goods has become a source of controversy  (for an overview, see Genetic Literacy Project, 2021). One side argues that GE contributes to making our situation worse but the other side claims that GE is key to overcoming societal challenges.^1^

More specifically, non-governmental organizations (NGOs) concerned about GE foodswarn of potential health damages related to GE (GM Watch, 2020), such as those caused by toxins or allergen substances (Garden Organic, 2021; Debating Europe, 2021). GE proponents, on the other hand, argue that, through GE, healthier foods can be developed, such as the famous golden rice (Golden Rice Project, 2021). Recent research shows that GE innovations can address vitamin deficiencies and play an important role in the defeat of famines and diseases (Rauner, 2017; Kohli & Dupont-Inglis, 2020). More recently developed traits include increased lycopene content in tomatoes (Wang et al., 2019; Ku & Ha, 2020). Heidi Godman of Harvard Medical School argues that these new properties bring health benefits, such as reduced risks of cancer or stroke (2012). By contrast, NGOs often report that GE crops are detrimental for the environment, for example through the risk of contamination or increased pesticide use (Friends of the Earth Europe, 2021), which harm animals, plants, and the ecosystem as a whole (Testbiotech, 2021; GeneWatch, 2021). GE supporting organizations retort that the use of GE has been found to reduce pesticide use (GMO Answers, 2020). Additionally, GE is said to provide further environmental benefits, such as products with longer shelf life to reduce waste (Debating Europe, 2021), increased soil-compatibility of crops (Parrott, 2018), and protection of biodiversity (Bayer, 2021).

Generally, the European GE debate revolves around economic risks and benefits. Opponents of the technology worry about economic consequences of its products, such as high costs, which are said to affect small farmers, particularly in poor countries, who suffer disproportionately (Cotter et al., 2015). This argument is often accompanied by a worry of large corporations having too much power (Voelker, 2020). On the other side, research reports higher crop yields through GE, which particularly benefits small farmers in developing countries (Klümper & Qaim, 2014).

Similarly, the European public is divided on the topic. In a special edition of the Eurobarometer, published by European Commission (2013), 61% of participants felt uneasy about GE foods. Although a decade later this number has dropped significantly, another Eurobarometer on Food Safety in 2019 showed that 27% of participants still see GE for food production as their main topic of concern (European Commission, 2019).

Generally, the positions taken in the public debate either favor or reject GE. Because the outcomes that both sides expect from this technology seem diametrically opposed and, thus, appear to be mutually exclusive. For example, as mentioned above, although proponents proclaim health benefits from GE foods, the opposition expects GE foods to be detrimental to human health. Consequently, the demands regarding this technology appear similarly incompatible: GE opposition calls for a continued ban on GE, or at least an extremely strict regulation (Die Grünen/EFA, 2016), but proponents call for a widespread approval of GE, or at least more open regulations (Albert, 2020).

These opposing positions have not yet been bridged in the European debate, causing a lack of regulation of state-of-the art technologies, to society’s detriment. Apart from the fact-based dissent, GE-positions appear to be based on deeply held beliefs. Regarding GE opponents, these beliefs have been described as morally absolutist (Scott et al., 2016; Fernbach et al., 2019). These findings indicate that the debate may also fail due to irreconcilable ideological divisions. It is important to note that the term “moral absolutism” is used in different contexts. Importantly, it can also be used in distinction to moral relativism (Gowans, 2021) to describe the view that there exists non-subjective moral truth independent of time and circumstances. This is not the way that moral absolutism is understood here. In our context, it describes a categorical conviction that GE is intrinsically morally good or bad irrespective of its specific consequences. In this sense, moral absolutists with respect to GE are chronically insensitive to empirical evidence concerning its actual effects.

It is the aim of this article to provide research that helps to understand better what causes this discourse failure regarding GE. We investigate moral attitudes relating to GE for food production. Our main objective is to shed light on the potential role of moral absolutism and principle or outcome-based moral attitudes in preventing constructive debates on GE.

This article proceeds as follows. In “Perception as Tradeoff within the Debate on Genetic Engineering”, we elaborate on the perception as a tradeoff that characterizes the European GE debate and the problems that come with it. In “Moral Absolutism Aggravates Perception as Tradeoff”, we explicate the concept of moral absolutism, which might constitute an obstacle for a fruitful debate. “Study Design” outlines the design of our representative empirical study to investigate moral absolutism of GE supporters and opponents with a representative German sample, and “Results” discusses the results and concludes the article.

## Perception as Tradeoff Within the Debate on Genetic Engineering

**Fig. 1:**
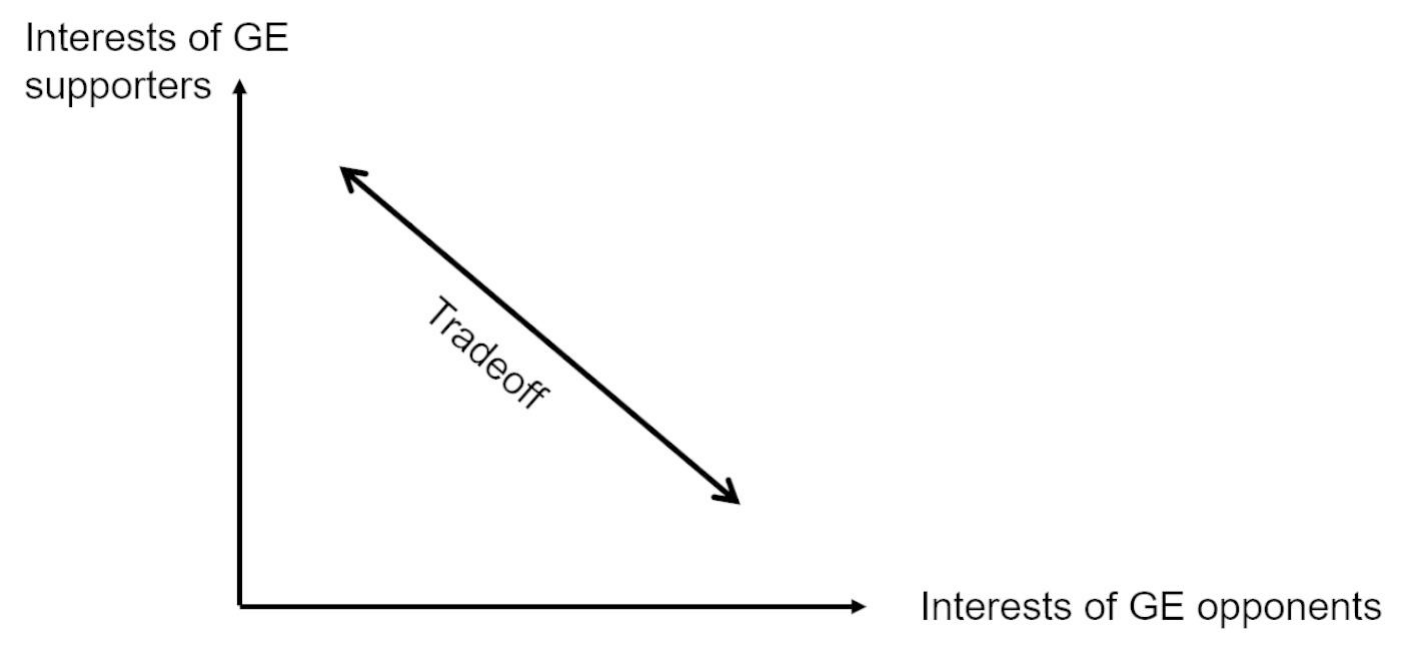
Interests in the GE debate are perceived as diametrically opposed (perception as tradeoff) (adapted from Pies et al., 2021)

Figure 1 illustrates the juxtaposed demands of GE opponents and supporters. The axes show the debating parties’ interests. For example, GE opponents *demand a ban* of GE because it could be a risk to human health. GE supporters *demand approval* of GE because they hold that GE could be beneficial to human health. Although both sides seem to officially share the common ends of human health and environmental integrity their positions on GE technology seem intransigent. This may be due to fundamentally opposed beliefs about the actual health effects of GE or because one or both groups only use this argument to cover up their absolutist attitude toward the technology. Consequently, potential debate outcomes are only thought of as being located along the graph. It thus appears that if the debate moves in the direction of the interests of one party, it necessarily moves away from those of the other party. The situation is perceived as a problem in which one party can only be better off at the expense of the other. This perception of a tradeoff is dominating the debate (Pies et al., 2017, 2021). Consequently, the public debate is conducted as if, ultimately, the winner-takes-all. This perception impedes the development of mutually beneficial agreements and, thus, leads to a blocked debate (Pies, 2009). As long as the debate revolves around such perceived tradeoffs, solutions are hard to find.

### Emotionalized Debating Fosters Perceptions as Tradeoff

In addition to these opposing views, the controversial debate is characterized by emotional campaigning on both sides. For example, Cotter et al. (2015) accused GE manufacturers of deliberately putting farmers into a dependency that forces them to buy ever more expensive seeds with ever more expensive pesticides, which ultimately drives them into ruin. Relatedly, using pejorative names and imagery, such as of “Frankenfood” and “fish tomatoes,” has been a common tool in anti-GE campaigning and for the media to grab attention (see, e.g., Hellsten, 2003; Spiegel International, 2009; GMO Awareness, 2021).

Conversely, on the support side, strong wording is used at times. In 2016, 110 Nobel laureates called on Greenpeace in a public letter to refrain from campaigns against GE (Roberts, 2016). Currently, 158 Nobel Laureates have signed. In this letter, the signatories charge anti-GE organizations with “denying the facts” and “misrepresenting their risks, benefits, and impacts.” They ask Greenpeace specifically to “cease and desist in its campaign against Golden Rice.” In this context, they call Greenpeace’s campaigning against GE in general and against Golden Rice in particular a “crime against humanity” (ibid.).

This perception of a tradeoff is exacerbated by debating strategies that are not aimed at finding common ground, but at portraying the opponent as someone who is acting in bad faith. Hence, debating is not meant to convince one’s opposition by arguments but aim at a third-party audience that the speakers intend to persuade. For example, molecular biologist Bock (2015, p. 4) speaks in one of his essays of “systematic self-deception, hypocrisy and mendacity […], which unfortunately seems to become a habit in our political landscape and for which the handling of the topic ‘genetic engineering’ has become almost symptomatic” (own translation). The geneticist Nellen (2018) spoke of “hysteria” and “ignorance” in one of his articles. Similarly, Szibor (2013) complained that all fears and frustrations of this world would be projected into green GE. These examples show that some scientists argue emotionally and, thus, sharply criticize the behavior of anti-GE organizations.

### The Consequences: Discourse Blocks Distort Policy-making

Such obstacles to a constructive debate are problematic because they may have adverse effects on policy-making. The legislation on GE in Germany^2^ is a case in point: This law restricts GE research and development with almost prohibitively high regulations and safety requirements which make it nearly impossible to develop GE products (Leopoldina, 2019, 2020). This legislation has been passed in 1990. Since then the regulations on cultivation and distribution of GE plants for human consumption have not been significantly revised to account for more recent scientific evidence and newer technological developments. Additionally, a more recent ruling by the European Court of Justice (ECJ) on the recently developed and more advanced genome editing technology (CRISPR/Cas9) in summer 2018 decided to regulate this new technology as strictly as the earlier GE technologies (Callaway, 2018). As a consequence of those policies, apart from one genetically modified potato plant (MON18), GE crops are virtually nonexistent in the European Union (Die Bundesregierung, 2021). Applied research and industrial research in biotechnology are leaving the European Union. For example, in 2012, BASF moved its GE unit into the United States (Zeit online et al., 2012).

According to the German National Academy of Sciences Leopoldina, the European Union’s regulatory framework on GE can not be scientifically justified (Dederer, 2020). Thus, they demanded a thorough renewal of this framework (Leopoldina et al., 2019). The Academy also called for European legislation on GE to be updated to reflect the current state of scientific opinion.

The failure of the legislative process to keep up with the scientific consensus is driven—at least in part—by a distorted public debate. Dysfunctional debates have consequences for policy making, as well as for individual behavior.^3^ According to Pies (2009), public debates influence policy-making by putting pressure on policymakers to placate public opinion. Ideally, discourse is a competition of ideas, in which the idea that creates the most appropriate balance between various stakeholder demands wins. However, distorted debates are reflected in the legislative process.This means that adopted policies may result in institutional frameworks that aredetrimental to society (Pies et al., 2017). As a consequence, innovations might be hampered and, thus, cannot be used to society’s advantage (van Eenennaam et al., 2021; Pies et al., 2017).

## Moral Absolutism Aggravates Perception as Tradeoff

### The Obstacle: Debating Parties See GE as a Goal, Not as a Potential Means

So far, the GE debate has not transitioned from perception as tradeoff towards an open-ended search for solutions that make all sides better off. On the contrary, as described earlier, GE supporters and opponents defend their own positions with emotional arguments. Moreover, their argumentation strategies aim to convince others (often third parties) of their own positions instead of contributing to finding mutually accepted solutions. Thus, instead of being perceived as a potential means to reach a common goal, GE is handled as the main goal itself. Banning or approving GE has become the central interest within the debate. This tendency is reflected in the recent European Court of Justice (ECJ) ruling on GE. For present regulation, the focus is on the technology used for breeding rather than the product. This is also reflected in the recent European Court of Justice (ECJ) ruling with respect to GE (Leopoldina et al., 2019, p. 5). In this regard, GE is not treated as a means for certain outcomes—or products—but as the central topic of interest. Here then, a means is normatively elevated to a moral end in itself (Pies, 2017). For one party, a *prohibition* of GE for food production has become the debate’s central moral goal, and for the other party, it is an *approval* of GE. Therefore, the result is a moral conflict. The problem with conflicting moral goals is that finding a compromise is even more unlikely because such moral goals can result in strong moral convictions that additionally impede constructive debates, as explained in the following section.

### Absolute Moral Convictions May Lead to Failed Debates

The drivers of discourses failure can also be studied on a psychological level by looking at discourse participants’ mindsets. Research in moral psychology investigates which cognitive mechanisms and mental models cause people to hold their beliefs as absolute, thereby shutting them off against new information and against opposing views. Beliefs about right or wrong can become *moral convictions*, such as abortion is wrong; nuclear energy is dangerous and should be prohibited; or genetically modifying organism for food production is wrong. Moral convictions are defined as “the subjective belief that something is *fundamentally* right or wrong” (Bauman & Skitka, 2009; italics added).

The concept of moral convictions investigated in the psychological literature can be linked to the philosophical concept of moral absolutism (see, e.g., Jackson & Smith, 2006; Rachels, 1970). Moral absolutism is the view that holds some actions are intrinsically right or wrong irrespective of their consequences (McConnell, 1981). In other versions, the absolutism does not apply to all but to some actions, which should be absolutely prohibited (Rachels, 1970). An example is the reference to divine laws that condemn certain kinds of behavior under all circumstances. In this sense, moral absolutism is an extreme form of nonconsequentialism, because consequences are strictly disregarded. This is not true for most nonconsequentialist theories. Whereas most nonconsequentialist views hold that “the moral status of an action is not determined solely by its consequences,” the absolutist “maintains that certain actions are *always* wrong, regardless of the consequences of not performing them” (McConnell, 1981, p. 287). McConnell (1981), thus, distinguishes between nonconsequentialist moral theories that are absolutist (complete disregard of consequences) and others that merely reject the exclusive consideration of consequences when assessing a given action. Specifically, one could say that an agent S is a moral absolutist with respect to an action A if and only if S holds that A is right (or wrong), and that the rightness (or wrongness) of A is entirely independent of the consequences of A.^4^

In the context of new technology evaluation, moral attitudes play an important role. If a technology is rejected solely on principled grounds, it is much harder to engage holders of this view in the debate. Fact-based arguments on the value of a technology only speak to people who do not reject the idea that a technology’s value is at least co-determined by its consequences. Misselhorn, for instance, argued that, as one cannot rule out that autonomous cars might run into moral dilemmas, the technology should be considered with moral skepticism because dilemmatic decisions should not be taken by automata irrespective of the benefits that they may generally bring in terms of traffic safety (Dörhöfer, 2018).

To study people’s moral views, the famous trolley case (Foot, 1967) has been used in empirical studies to find out whether participants prefer a rule-based (i.e., nonconsequentialist) or an outcome-based (i.e., consequentialist) moral approach. The thought experiment reveals a dilemma between good consequences (e.g., saving five human lives but causing one person to die) and a profound moral principle (e.g., no act is permissible that causes harm or kills a human being). Participants who find the act that saves the lives morally permissible are thus identified as outcome-minded, whereas those who find the act impermissible are identified as rule-minded. This way of distinguishing participants in behavioral experiments has revealed systematically different behavioral patterns and moral attitudes (Cornelissen et al., 2013; Ostermaier & Uhl 2017).

Thus far, empirical research has investigated the moral attitudes of GE opponents. Scott et al. (2016) found that roughly 70% of people with anti-GE beliefs qualify as moral absolutists. Furthermore, Fernbach et al. (2019) showed that extreme GE opponents tend to think they know more about the GE foods than others do but, on average, know less when tested on genetics.

## Study Design

We preregistered our hypotheses, measures, and planned analyses at https://aspredicted.org/xn24p.pdf.

For data collection, we conducted an online survey through the German online panel provider GapFish. Our sample is representative of the German population according to age, gender, income, and education. After sorting according to an attention check, our analysis includes complete responses from 636 participants.

The study took approximately 15 min to complete. After providing informed consent and demographic information, participants indicated their overall attitudes toward GE for food production (see the Measures section). Depending on their responses, participants received one of two versions of the questionnaire—one tailored to GE opposition, and the other tailored to GE support. First, they were asked to select an NGO to which they may want to donate. This NGO either supports the ban of GE plants or the admission of GE plants, respectively. Second, participants had to state whether they would like to donate or not.

In the second part of the survey, participants had to evaluate items on moral absolutism regarding GE (described below). We also asked participants to which degree they generally find NGO activities and donating to be effective. In addition, participants had to indicate how important they find spirituality and religion for their personal lives and were asked to rate the standard trolley case morally (Foot, 1967). The survey continued with three questions of the standard cognitive reflection task (Shane, 2005), an elicitation of participants’ political orientation, and three exploratory open questions about naturalness and sanctity. The study ended with an attention check and the opportunity to give feedback.

Below, we describe our main measures in more detail.

### Main Measures

#### GE Attitude

Participants were asked to indicate their attitude toward GE (“Which statement is closest to your position toward genetically engineered plants for human consumption”) on a six-point Likert scale, anchored by “I am strongly against it” and “I am strongly in favor of it.” Participants who selected one of the first three Likert points (i.e., ranging from “I am strongly against it.” to “I tend towards opposing it.”) were subsequently provided the survey version for GE opponents. Respectively, participants who selected one of the last three Likert points (e.g., ranging from “I tend towards supporting it.” to “I am strongly in favor of it.”) were subsequently provided the survey version for GE supporters.

#### Moral Absolutism

Moral absolutism was assessed adopting three agree/disagree statements from previous research (Baron & Spranca, 1997; Scott et al., 2016). For GE opponents, these were as follows. (a) “The GE of plants for human consumption should be prohibited. This should apply regardless of how great the benefits and how small the risks of genetic engineering are” (b) “If the genetic engineering of plants for human consumption is only approved on a restricted basis, this is just as wrong as unrestricted approval. The extent of the approval does not matter.” (c) “Approval of genetic engineering of plants for human consumption would be wrong even in a country where everyone thinks approval would be right.”

For GE supporters, these were as follows. (a) Genetic engineering of plants for human consumption should be allowed, regardless of how great the risks and how small the benefits of genetic engineering.” (b) “If the genetic engineering of plants for human consumption is partially banned, this is just as wrong as a complete ban. The extent of the ban does not matter.” (c) “Banning genetic engineering of plants for human consumption would be wrong even in a country where everyone thinks the ban would be right.”

#### Willingness to Donate

Participants were asked to select one NGO to which they would potentially donate. For GE opposition, they could select from the following German NGOs: *Gene-ethical Network, Interest Group for GE-free Sowing, GE-free Regions*, and *Alliance for GE-free Agriculture.*^5^ For GE support, subjects could select from the following German NGOs: *Transparency Genetic Engineering, World Health Organization, Forum Green Rationality*, and *Innoplanta*.^6^ Participants could also select “I find them all equally bad” or “I find them all equally good,” respectively. Participants then had to select, hypothetically, whether they would like to donate 5Euros to their previously selected NGO.

#### Trolley Problem

This measure assessed whether participants decided based on consequentialist or rule-based theory. Describing the standard version of the trolley problem, participants were given a scenario in which five workers would die through an approaching train if no action was taken (i.e., changing the switch) versus one worker would die if action was taken. Participants then had to select whether they found it morally acceptable to change the switch.

#### Cognitive Reflection Task

Three cognitive reflection tasks were adapted from Shane (2005). These tasks recorded whether participants tended to reflect on given problems or rather decided intuitively. The first one read, “A racket and a ball cost a total of 1.10 Euros. The racket costs 1.00 euro more than the ball. How much does the ball cost?” The second read, “If it takes 5 machines 5 minutes to make 5 devices, how long would it take 100 machines to make 100 devices?” The third read, “In a lake there is a small area covered with lily pads. Every day the area doubles. If it takes 48 days to cover the entire area of the lake with water lilies, how long would it take to cover half the area of the lake with water lilies?” Participants then had to provide their solutions in free text boxes.

## Results

### Main Results

Of our 636 survey participants, 484 (76.1%) can be identified as holding at least moderately negative attitudes toward GE (i.e., moderately oppose, oppose, or strongly oppose). A minority of 152 (23.9%) can be identified as holding at least moderately positive attitudes toward GE (i.e., those who moderately support, support, or strongly support). In the following, we refer to the former group as “opponents” and to the latter as “supporters.” It is noteworthy that opponents express their preference against GE more strongly than supporters express their preference for GE. The median answer of the opponents is that they *oppose* GE, but the median answer of the supporters is that they *moderately support* them. Panel 1 of Table 1 provides an overview of the numbers and proportions of participants that *moderately* oppose (or support), oppose (or support) with *medium* intensity, and *strongly* oppose (or support) the GE of crops for human consumption.

We first investigate whether supporters and opponents show different levels of agreement with statements that express moral absolutism. It turns out that opponents agree on average with 2.29 (SD = 1.02) of the three absolutist statements, but supporters agree on average with only 1.42 (SD = 1.05) of the three absolutist statements. This difference is statistically highly significant (*p < 0.001*, M.W.U Test). One might suspect that this effect is merely driven by the fact described above: the *opponents’ preference against GE is stronger than the supporters’ preference for GE*. Therefore, we compare the agreement with the absolutist statements for each of the three given levels of preference intensity separately. This demonstrates that we observe a highly significant difference in the agreement with the absolutist statements between opponents and supporters for each of the three levels of preference intensity. The results are summarized in Panel 2 of Table 1.

**Table 1:** Preferences against and for GE and corresponding agreement with absolutist statements

	Panel 1: numbers (proportions) of participants by preference		Panel 2: means (standard deviations) of degree of moral absolutism by preference		
Preference intensity	Against GE Crops	For GE Crops	Against GE Crops	For GE Crops	difference in means (M.W.U test)
Moderate	203 (31.9%)	103 (16.2%)	1.97 (1.13)	1.44 (1.01)	*p < 0.001*
Medium	131 (20.6%)	35 (5.5%)	2.39 (0.89)	1.37 (1.06)	*p < 0.001*
Strong	150 (23.6%)	14 (2.2%)	2.65 (1.43)	1.43 (1.34)	*p < 0.001*
All	484 (76.1%)	152 (23.9%)	2.29 (1.02)	1.42 (1.05)	*p < 0.001*

The literature suggests that moral absolutism is related to nonconsequentialist or deontological ethical thinking. Therefore, we check whether GE opponents and supporters express a different willingness to pull the lever in the traditional trolley problem. Pulling the lever implies the outcome-based choice of deviating the trolley to the sidetrack and, thus, intentionally killing one person to save five. Not pulling the lever implies the principle-based choice of refusing to kill a person intentionally, irrespective of some greater good. Indeed, we find that only 322 of 484 GE opponents (66.5%) are willing to pull the lever, but 120 of 152 supporters (78.9%) are willing to do so (*p* = 0.003, Fisher’s Exact Test). This result suggests that opponents are indeed less likely to make an outcome-based choice in the trolley dilemma than supporters are.

One might expect that a higher conviction for one’s cause also reflects a higher commitment to make personal sacrifices for one’s cause. Therefore, we offered opponents a list of NGOs that were committed to preventing GE, and supporters were offered a list of NGOs that were committed to promoting GE. We asked either group to choose their preferred NGO. Later in the survey, we asked participants for their willingness to donate 5 Euros for the NGO that they had previously selected. We find that 168 of the 484 (34.7%) opponents state a willingness to donate to their cause of preventing GE, while only 37 of the 152 (24.3%) supporters state a willingness to donate to their cause of promoting them (*p = 0.017*, Fisher’s Exact Test). This implies that opponents of GE express a higher commitment to their cause in monetary terms than supporters do. Notice that this effect could also be based on opponents’ relatively stronger trust in the efficacy of “their” NGOs. Therefore, we asked opponents (supporters) whether they believed, first, that NGOs lobbying for banning (promoting) GE were effective and, second, whether they considered small donations to these GE to be effective. Participants expressed their approval rating to the statement that NGOs committed to their respective causes are effective and that small donations would be effective on Likert scales from 1 (fully agree) to 7 (do not agree at all). Indeed, opponents have a greater trust in the efficacy of “their” NGOs than supporters do (3.05 vs. 3.37, *p* = 0.009, M.W.U. Test), as well as in the efficacy of small donations to their respective cause (3.36 vs. 3.72, *p* = 0.012, M.W.U Test). This greater trust can also be explained against the background of the current regulations in the European Union. An almost complete ban on GE for food production can be interpreted as a success of anti-GE NGOs.

Finally, we checked for potential differences in the levels of cognitive reflection shown by opponents and supporters of GE. To this aim, we compared the correct solutions to the three cognitive reflection tasks. The average number of tasks that were correctly solved by opponents and supporters were similarly low (0.86 vs. 0.82, *p = 0.393*, M.W.U. Test). This suggests that both groups do not differ in their levels of cognitive reflection.

### Analysis of Demographic Differences

In our representative German sample, opponents are on average approximately 5 years older than supporters (45.0 vs. 39.8, *p < 0.001*, M.W.U. Test) and more likely to be female (55.4% vs. 39.5%, *p < 0.001*, Fishers’ Exact Test). In terms of political preferences, fewer of the opponents are willing to vote for one of the parties currently represented in the German Bundestag (24.5% vs. 12.5%, *p = 0.002*, Fisher’s Exact Test).

Participants were also asked for their agreement to the statements that spirituality and religion play an important part in their lives (1 = fully agree, 7 = do not agree at all). Participants tend to ascribe a generally low importance to spirituality and religion. The importance that opponents and supporters ascribe to spirituality does not differ significantly (4.72 vs. 4.54, *p = 0.266*, M.W.U. Test). Opponents, however, assign an even lower importance to the role that religion plays in their lives than supporters do (4.89 vs. 4.47, *p = 0.039*, M.W.U Test).

## Discussion and Conclusion

Our study investigates the prevalence of absolutist moral attitudes in GE supporters and opponents that may contribute to the failed public debate on this technology. We find that both camps of our representative German sample show moral absolutism in their GE attitudes, yet moral absolutism is more pronounced with opponents than supporters. This effect remains if we control for the strength of the conviction. These findings are further strengthened by participants’ answers to the trolley problem. Here, we find that GE opponents are more likely to give a principle-based answer than GE supporters are. In the literature, some principle-based (or nonconsequentialist) moral views have been described as absolutist, which is in line with what we find in our data. GE opponents also exhibit a higher willingness to donate 5 euro to their cause compared to GE supporters. This effect might be driven by stronger trust in the effectiveness of those NGOs, which might be attributed to the successful anti-GE campaigns of NGOs. It remains an open question whether the lack of willingness to donate by GE supporters is mainly driven by their lack of trust in the respective NGOs’ effectiveness to change current regulation. Other reasons, such as the belief that a change in regulation has very poor chances of success in Europe or that lack of interest in the topic to consider donation a viable option, might also play a role.

Our findings may inspire more empirical inquiries into questions on the ethics of technology. Moral intuitions have the adaptive advantage to detect violations of moral values in social behavior (e.g., if a perpetrator harms someone). Yet, morality may also play an ambivalent role. Greene (2014) argues that although our moral intuitions are well-equipped to react to issues that have been familiar to humanity for a long time, this adaptation cannot be assumed regarding technologies that emerged only in the most recent human history (Greene, 2014). If Greene is correct, it would be worthwhile to investigate more systematically whether absolutist or consequentialist responses of participants with regard to technologies are asymmetrically based on intuitive or deliberative reasoning.

Relevant implications for the ethics of technology arise if our cognitive mechanisms have evolved to focus on harm that might be caused by something happening instead of harm that might be caused by this something being prevented from happening. This phenomenon has been described as omission bias (Baron & Ritov, 2004). It should be noted that evolution need not be understood only genetically. For instance, Gintis (2011) and Gintis et al. (2012) suggest that a gene-culture coevolution determines basic principles of human morality. In any case, this cognitive mechanism would lead to a skepticism that may transcend the necessary caution toward new technology and may inhibit societal benefits that can be brought about by the introduction of that technology. Our findings suggest that ethical research accompanying new technologies should consider this understated aspect by stressing the point that engaging, as well as forgoing, a technology has far reaching societal consequences and the potential of harm (Deutsch, 2011).

**Fig. 2:**
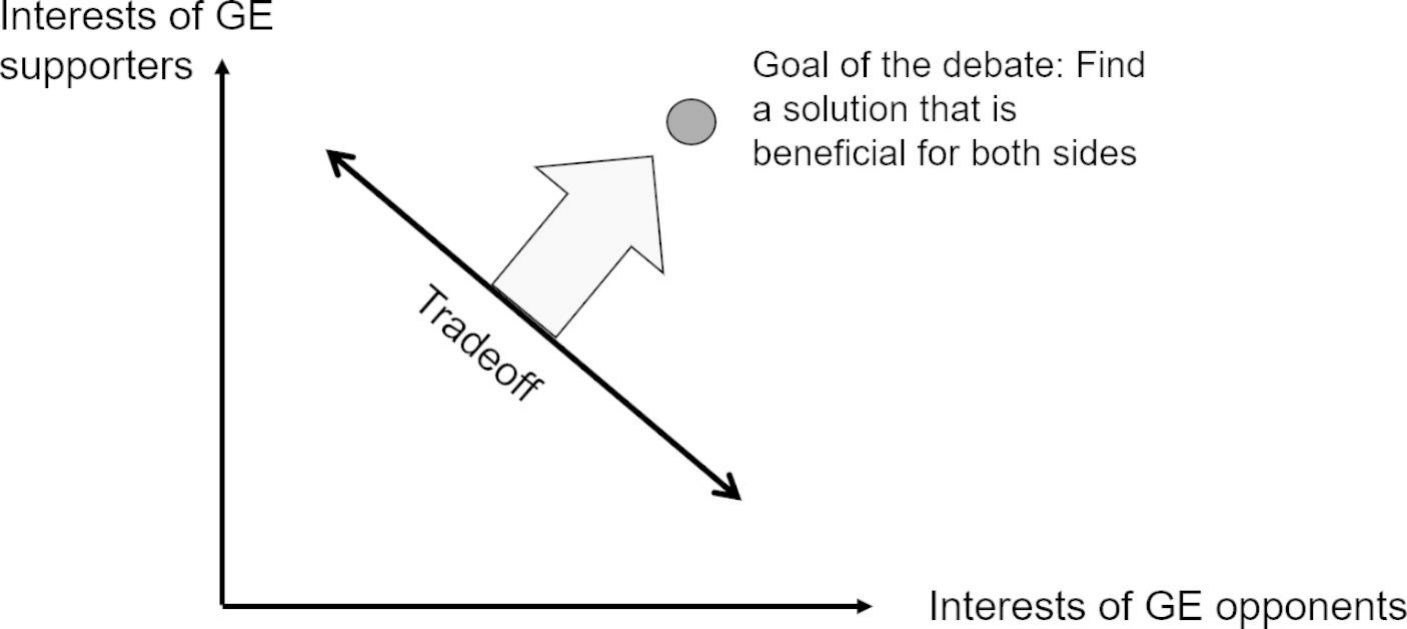
A beneficial solution lies outside the perception as a tradeoff (adapted from Pies et al., 2021)

In a democratic society, open debate is the prerequisite to successful regulation, and failed debates should be prevented. Our findings indicate that moral absolutism is one driver that complicates GE debates because this moral attitude leads the debating parties to perceive attitudinally dissimilar others as adversaries (i.e., the interests of the debating parties are perceived within a tradeoff). This empirically supported diagnosis implies that such debating failures can be overcome if the perception is changed, and thus, the issue is no longer perceived within a win–lose paradigm but as a search for solutions of mutual improvement (Pies, 2009). Technically speaking, the tradeoff line—according to which one party’s win is the other one’s loss—is abandoned; instead, both parties search for solutions that make them both better (see Fig. 2). The goal of the debating parties is no longer to realize predetermined goals at the expense of other parties, but rather to engage in a search for a new goal that can be shared by the other party. Naturally, this is a tedious process in which, at times, only incremental improvements might be achieved, but in a liberal democracy, it remains the way to achieve societal progress. Within the GE debate, this paradigm shift would mean that the debating parties no longer focus on mere means, i.e., banning or admitting GE crops to the European market, but widen their perspective to the *goals* that should be reached by these means.

Specifically, there is a divide between those who focus on the effects on the ecosystems and those who focus on farming efficiency. Consequently, the interests of these two types of agriculture—conventional and organic—appear to clash. On the one hand, organic agriculture is more concerned with ecological farming methods but lacks efficiency (Meemken & Qaim, 2018, p. 57). Conventional farming, on the other hand, is more efficient but lacks adaptive farming methods (Leopoldina et al., 2019, p. 11). However, considering the ever-increasing necessity for sustainable production, both types of agriculture need to move toward one another; conventional agriculture needs to become more ecological, and organic farming needs to become more efficient (Pies et al., 2021). Focusing on the common goal to make agriculture more sustainable opens up room for mutual betterment. For these purposes, GE can be a means to reach a common goal: GE opens up opportunities for both, for example, more ecological farming through reduced pesticide use and more efficiency through higher yields (Ahmed et al., 2020, p. 1).

## Footnotes

1 Naturally, each side uses such arguments that make their respective position stronger. That is, proponents of GE tend to focus on potential benefits, while opponents of GE tend to focus on potential risks. However, such generalized judgments of all products of a technology are questionable. At the same time, proponents of GE that are most active in the German debate are often scientists involved in the technology (Pies et al., 2021). This is because the vast majority of scientific evidence supports the claim that GE products have generally favorable traits, are as safe as conventional crops and that potential risks are not inherent in the technology (see, for example, Leopoldina et al., 2019; NASEM, 2016; BBAW, 2018; BMBF, 2014). Opponents of GE involved in the German debate are mostly NGOs (Freitag, 2013). While these NGOs also claim to base their arguments on scientific evidence, some research suggests that these studies made by NGOs show systematically biased results that seem to skew their findings towards their position of opposition (Klümper & Qaim, 2014).

2 https://www.gesetze-im-internet.de/gentg/index.html. While there is no English translation of the German law, please consult the following page for some information in English: https://www.bvl.bund.de/EN/Tasks/06_Genetic_engineering/02_Consumers/07_Legal_Framework/01_Germany/Germany_node.html.

3 The detrimental influence public images or “agrarian myths” can have on agricultural policy has been illustrated by Browne et al. (2019) in their book, “*Sacred cows and hot potatoes: agrarian myths and agricultural policy.”*

4 We are grateful to an anonymous reviewer for helping us to clarify this point.

5 Original names: Gen-ethisches Netzwerk e.V., IG Saatgut: Interessengemeinschaft für gentechnikfreie Saatgutarbeit, Gentechnikfreie Regionen, and Bündnis für gentechnikfreie Landwirtschaft.

6 Original names in German: TransGen (Transparenz Gentechnik, Forum Bio- und Gentechnologie e.V.), Weltgesundheitsorganisation, Forum Grüne Vernunft e.V., and Innoplanta e.V.
